# 658. Oral Vancomycin Taper for First Occurrence of *Clostridioides difficile* Compared to Standard of Care: An Observational Retrospective Matched Cohort Study

**DOI:** 10.1093/ofid/ofad500.721

**Published:** 2023-11-27

**Authors:** Sarah E Moore, Elena A Swingler, Matthew Song, Ashley M Wilde

**Affiliations:** Norton Healthcare, Louisville, Kentucky; Norton Healthcare, Louisville, Kentucky; Norton Healthcare, Louisville, Kentucky; Norton Healthcare, Louisville, Kentucky

## Abstract

**Background:**

*Clostridioides difficile* infection (CDI) recurrence rates may be reduced with fidaxomicin, but financial barriers can limit access. Tapered and/or pulsed oral vancomycin is recommended in patients with recurrent CDI, however there is currently limited data for use in treating initial CDI.

**Methods:**

Multicenter retrospective propensity-matched cohort study in patients ≥ 18 years old hospitalized with a first occurrence of CDI from 6/18/2018 – 12/21/2021 conducted at Norton Healthcare in Louisville, Kentucky. Standard of care (SOC) was receipt of oral vancomycin for 10-14 days compared to a standardized oral vancomycin taper (Figure 1). Subjects were matched based on age < or ≥ 65 and receipt of non-CDI antibiotics during index hospitalization or within 6 months post-discharge. Selected baseline characteristics and receipt of CDI and non-CDI antibiotics were collected. Non-CDI antibiotics were stratified by high, medium or low risk for CDI. The primary outcome was CDI recurrence within 6 months of hospital discharge. Secondary outcomes included time to recurrence, recurrence rates stratified by age group < or ≥ 65 years, CDI severity of illness per IDSA guidelines and all-cause mortality within 6 months of hospital discharge. Categorical variables were analyzed using Chi-squared and Fisher’s exact tests as appropriate.

Standardized Oral Vancomycin Taper

**Figure:**
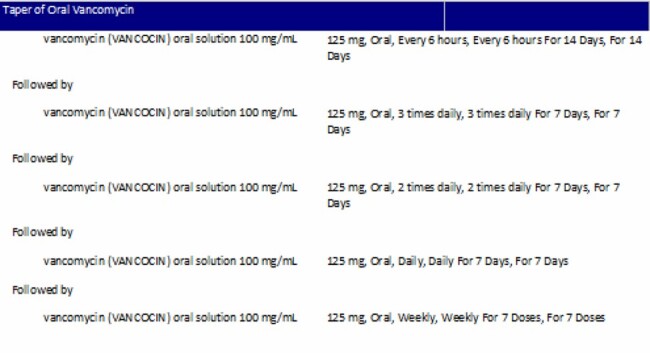

**Results:**

Recurrence rates at 6 months were 5.3% (4/75) in the taper arm and 28% (21/75) in the SOC arm (p=0.002). All-cause mortality was 20% (15/75) in the taper arm and 5.3% in the SOC arm (p=.026). Severe/fulminant CDI occurred in 49.3% of the taper arm and 58.7% of the SOC arm (p=0.53). Both groups received antibiotics at the same rate based on matching, and rates of antibiotics considered high risk for CDI were similar at 65% in the taper and SOC groups (65% vs. 61.3%, p=0.809). More patients in the taper arm were immunocompromised (25.3% vs 9.3%, p=0.029).

**Figure:**
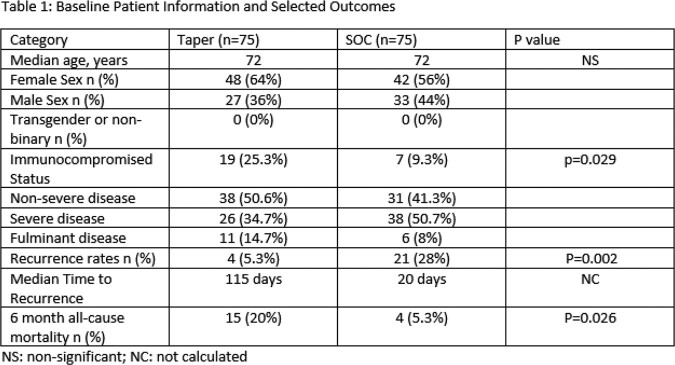

**Conclusion:**

A significantly lower rate of CDI recurrence was observed at 6 months with tapered oral vancomycin coupled with higher all-cause mortality in patients being treated for first CDI. Additional information is needed to elucidate the clinical utility of this approach.

**Disclosures:**

**Ashley M. Wilde, PharmD, BCIDP**, Pfizer: Grant/Research Support

